# Chronic Overexpression of Bradykinin in Kidney Causes Polyuria and Cardiac Hypertrophy

**DOI:** 10.3389/fmed.2018.00338

**Published:** 2018-12-03

**Authors:** Carlos C. Barros, Ines Schadock, Gabin Sihn, Franziska Rother, Ping Xu, Elena Popova, Irina Lapidus, Ralph Plehm, Arnd Heuser, Mihail Todiras, Sebastian Bachmann, Natalia Alenina, Ronaldo C. Araujo, Joao B. Pesquero, Michael Bader

**Affiliations:** ^1^Department of Nutrition, Federal University of Pelotas, Pelotas, Brazil; ^2^Department of Biophysics, Federal University of São Paulo, São Paulo, Brazil; ^3^Max Delbrück Center for Molecular Medicine, Berlin, Germany; ^4^Charite-University Medicine, Berlin, Germany; ^5^Federal University of Minas Gerais, Belo Horizonte, Brazil; ^6^Berlin Institute of Health (BIH), Berlin, Germany; ^7^German Center for Cardiovascular Research (DZHK), Partner Site Berlin, Berlin, Germany; ^8^Institute for Biology, University of Lübeck, Lübeck, Germany

**Keywords:** kinin, kallikrein-kinin system, blood pressure, polyuria, rats

## Abstract

Acute intra-renal infusion of bradykinin increases diuresis and natriuresis via inhibition of vasopressin activity. However, the consequences of chronically increased bradykinin in the kidneys have not yet been studied. A new transgenic animal model producing an excess of bradykinin by proximal tubular cells (KapBK rats) was generated and submitted to different salt containing diets to analyze changes in blood pressure and other cardiovascular parameters, urine excretion, and composition, as well as levels and expression of renin-angiotensin system components. Despite that KapBK rats excrete more urine and sodium, they have similar blood pressure as controls with the exception of a small increase in systolic blood pressure (SBP). However, they present decreased renal artery blood flow, increased intrarenal expression of angiotensinogen, and decreased mRNA expression of vasopressin V1A receptor (AVPR1A), suggesting a mechanism for the previously described reduction of renal vasopressin sensitivity by bradykinin. Additionally, reduced heart rate variability (HRV), increased cardiac output and frequency, and the development of cardiac hypertrophy are the main chronic effects observed in the cardiovascular system. In conclusion: (1) the transgenic KapBK rat is a useful model for studying chronic effects of bradykinin in kidney; (2) increased renal bradykinin causes changes in renin angiotensin system regulation; (3) decreased renal vasopressin sensitivity in KapBK rats is related to decreased V1A receptor expression; (4) although increased renal levels of bradykinin causes no changes in mean arterial pressure (MAP), it causes reduction in HRV, augmentation in cardiac frequency and output and consequently cardiac hypertrophy in rats after 6 months of age.

## Introduction

The kallikrein-kinin system (KKS) is present throughout the body. Bradykinin (BK) is its main effective peptide and has two sources: it is released from circulating high molecular weight kininogen by the action of plasma kallikrein; or from low molecular weight kininogen by the action of tissue kallikrein (1). BK is an agonist of the kinin B2 receptor (B2R), and its metabolite Des-Arg^9^-BK has affinity to the kinin B1 receptor (B1R). First related to vasodilation, inflammation, pain, and edema, the KKS has also been shown to participate in several cellular and physiological events, such as glucose homeostasis (2–4), leptin sensitivity (5), and organ and tissue protection or injury (6). The effects of BK on renal physiology and blood pressure (BP) control are still not completely unraveled.

A number of experiments were carried out to understand the role of BK in kidney and in particular how it is involved in the control of renal homeostasis and BP, as previously reviewed (1, 7). Usual approaches to test intrarenal effects of BK are the renal artery infusion of BK or antagonists of its receptors (8–10), intrarenal tissue BK infusion (11), the use of knockout animals (12, 13), or *in vitro* studies (14, 15). However, all approaches have limitations, mainly due to the complex morphology of the kidney, where the proximal and distal parts of the nephrons are anatomically mixed, and the local circulation has very specific characteristics and morphology. More recent studies have shown that infusion of BK into the kidneys causes increased urinary volume and increased sodium secretion, with no change in BP (16). Studies in dogs showed that infusion of BK in the kidneys increases urinary volume (UV) and sodium excretion (UNaV), without changing glomerular filtration rate (GFR) and these effects are blocked by pretreatment with the B2R antagonist HOE-140 (17). The increase in UV is associated with vasopressin inhibition in the kidney. Schuster et al. has shown that lysyl-BK inhibits the effect of vasopressin with respect to hydraulic conductivity (Lp) in the rabbit cortical collecting tubule perfused *in vitro* (18). The inhibition of vasopressin activity by BK infusion was also confirmed in an *in vivo* study (17). Hebert et al. concluded that this effect is transmitted via B2R and is independent of calcium signaling in the cortical collecting ducts.

Both previous *in vivo* and *in vitro* experiments revealed several acute effects of BK in kidneys, but the models used are not predicting chronic effects of excess BK in this organ. This knowledge is important to evaluate the possibility of using B2R agonists for renal diseases.

Therefore, we generated a transgenic rat overexpressing BK in the proximal convoluted tubules, in order to test the hypothesis that chronic excess of bradykinin may cause changes in BP and physiology of the cardiovascular system. These animals were submitted to diets with different sodium contents. Changes in BP, heart rate variability (HRV), genetic expression of selected components of the renin-angiotensin, vasopressin, and KKSs, as well as cardiac morphology, and physiology of the cardiovascular and urinary systems were evaluated.

## Methods

### Animals

All studies were performed in accordance with the guidelines for the humane use of laboratory animals by the Max Delbrück Center for Molecular Medicine (Berlin, Germany) and with EU Directive 2010/63/EU for animal experiments. The animals were maintained under standardized conditions with an artificial 12-h dark-light cycle with free access to standard chow (0.25% sodium, SSNIFF Spezialitäten GmbH, Soest, Germany) and water *ad libitum*, except during the experimental diet period when they received special sodium containing diets. Two different diets were fed, low salt diet and high salt diet (E15430-24 sodium deficient; E15431-34, 4% salt, SSNIFF Spezialdiäten GmbH, Germany). If not stated different, male rats at the age of 4 months were used for experiments. All procedures with the animals were analyzed and approved by the Ethical Committee Landesamt für Gesundheit und Soziales (LaGeSo), Berlin, Germany.

### Generation of transgenic rats

**Figure 1:**
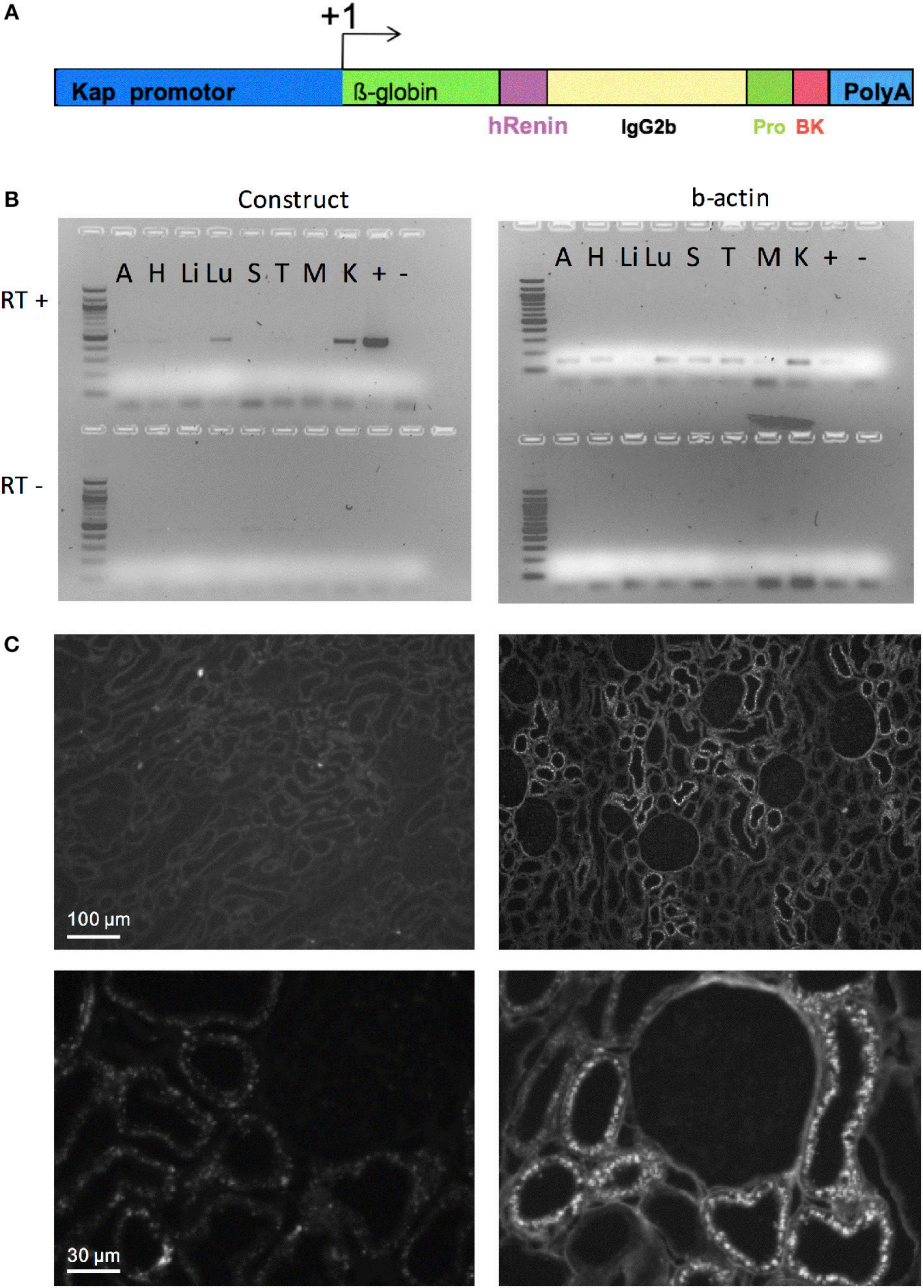
Targeted overexpression of BK in proximal convoluted tubules. **(A)** Construct for BK secretion in proximal tubule. The Kap promoter and an intron from β-globin was used to target renal proximal tubule expression of a recombinant protein containing: a signal peptide from human renin gene (hR) to allow the recombinant protein to be secreted; part of mouse IgG2b to provide mass; a human prorenin-derived spacer (Pro) to allow furin activity; a furin cleavage site, the sequence of the Tyr^0^-BK peptide (BK) followed by a stop codon and a polyadenylation site (polyA). **(B)** RT-PCR analysis of selected tissues of KapBK rats. Image of agarose gels showing the PCR products of β-actin (right) and mouse IgG2b (left) after reverse transcriptase reaction (RT+) of total RNA extract from KapBK rat tissues. As control for DNA contamination the same procedure was done with samples submitted to the same protocol without addition of reverse transcriptase (RT-, bottom of the gels). A, Adrenal gland; H, Heart; Li, Liver; Lu, Lung; S, Spleen; T, Thymus; M, Muscle; K, Kidney; +, positive control; – = H_2_O. **(C)** Immunohistochemistry using anti-mouse IgG2b antibodies to show the specific expression of the recombinant protein in kidney proximal tubules. Upper panels show overview of the cortex with immunoreactive signal over proximal tubules exclusively in the KapBK tissue. Lower panels show higher magnification with immunoreactive proximal segments grouped around a single glomerulus; magnification x 150 (top), x 800 (bottom).

The basic DNA construct for the generation of transgenic rats codes for an engineered fusion protein consisting of the signal peptide of human renin, the Fc portion of the mouse IgG, a prosegment of the human prorenin, a furin cleavage site followed by the sequence of Tyr^0^-BK, as previously described (19). The promoter of kidney androgen regulated protein (Kap) was used to direct the expression of this construct specifically in the proximal tubules (20). This peptide delivery system has already been successfully used in several transgenic animal models (21–23). Tyr^0^-BK, rather than BK itself, had to be selected, since furin would only very inefficiently cut N-terminal to the natural start of the BK peptide, arginine, but will cut in front of tyrosine (19). Tyr^0^-BK is known to be a full agonist on BK receptors (24). The Kap promoter sequence was cloned into the same vector and the resulting construct (Figure 1A) was linearized and microinjected into male pronuclei of Sprague-Dawley rat zygotes as previously described (25). Genomic integration of the transgene DNA construct was determined by PCR analysis using DNA obtained from offspring tail biopsies.

### Assessment of basic renal function

To collect urine, rats were kept in metabolic cages with 1 day of adaptation before collecting samples for analysis. Overnight collected urine samples were centrifuged (4,000 g), the supernatant collected in fresh tubes and stored frozen at −20°C. For measurement of urine concentrations of ions and creatinine, samples were sent and processed by Labor28 (Berlin, Germany).

### Immunohistochemistry

For histochemical analysis, rats were anesthetized with Nembutal (100 mg/Kg body weight), the abdominal cavity opened and kidneys were fixed by retrograde perfusion through the abdominal aorta using 3% paraformaldehyde in PBS as previously described (26). Kidneys were then processed for cryostat sectioning. Cryosections were incubated with primary anti-mouse IgG2b and secondary anti-rabbit Cy2-fluorescence-coupled antibodies and examined in a Leica fluorescence microscope.

### High frequency ultrasound analysis

Animals were anesthetized by inhaling a 2.5% isoflurane oxygen mixture in a heat-controlled chamber to keep the body temperature stable. After reaching full anesthesia, rats were fixed on a heated plate with electrodes on their paws to control heart and respiratory rate, as well as to record the electrocardiogram. Body temperature was monitored by a rectal sensor and corrected by a heating lamp if necessary. With the ultrasonic detector MS-250 collected data were visualized and analyzed via the VEVO 2,100 high-resolution imaging system (Visualsonics Fujifilm, VisualSonics, Toronto, Ca). Stroke volume and cardiac output was measured by tracing the endocardium in systole and diastole of a parasternal long axis view of the left ventricle.

### Telemetry

The telemetry system for BP and heart rate measurement (Dataquest ART 4.0™, Data Sciences Inc., St. Paul, MN, USA) and the implantation procedure are described in detail by Plehm et al. (27). Briefly, the radiotelemetric pressure transducers (TA11PA-C40) were implanted in the abdominal cavity of the rats, with the pressure-sensing capillary anchored in the lumen of the abdominal aorta. Before the implantation the zero offset was measured and the unit was soaked in 0.9% NaCl solution. Rats were anesthetized with isoflurane. Animals recovered for 10 days before baseline values were recorded. By this time the rats had regained their circadian BP and heart rate rhythm while surgery and anesthesia-induced changes in systolic blood pressure (SBP), diastolic blood pressure (DBP), mean arterial pressure (MAP), and heart rate (HR) had abated. The data from the implanted transducer were transmitted via radiofrequency signals to a receiver below the home cage and sampled every 5 min (sampling rate 500 Hz).

### Spectral analysis

Continous telemetric recordings of the BP waveform were stored during resting and activity periods for 1 h. These waveform recordings were used for computing the HRV and the spectral analysis of the HR to analyze the autonomic regulation. For analysis of the HRV and the frequency domain results we used the Kubios HRV analysis software (Kubios, Kuopio, Finland). The HRV spectrum was calculated from artifact-corrected 30 min recordings using Fast Fourier Transformation. The frequency bands were very low frequency (VLF, 0,015–0,250 Hz), low frequency (LF, 0,250–1,000 Hz), and high frequency (HF, 1,000–6,000 Hz).

### Measurement for renin-angiotensin system (RAS) components

The renin activity of plasma samples was calculated from the generation rate of AngI in the samples, for renin concentration a defined amount of angiotensinogen was added before incubation. Plasma angiotensinogen was measured by adding rat kidney extract to the samples and measuring AngI generation. The concentration of AngI was measured by radioimmunoassay. These methods were performed as described before (28, 29).

### Gene expression analyses

For quantitative real-time PCR (qPCR) tissues were snap-frozen in liquid nitrogen. Samples were homogenized and total RNA was isolated using a NucleoSpin RNA II purification kit (Macherey-Nagel, Düren-Germany) and then stored at −80°C until use. The RNA integrity was assessed by electrophoresis on an agarose gel. cDNA was synthesized from 1 μg of total RNA with Moloney murine leukemia virus reverse transcriptase (Promega, Madison, USA) using random hexamer nucleotides. Standard curves were made to determine the amplification efficiencies for each primer pair. Quantitative PCR was performed on an ABI Prism 7900 sequence detection system with 100 nM primers, 5 ng of cDNA, and 10 μL SYBRGreen mastermix (Thermo Fisher Scientific, Waltham, USA) in a 20 μL reaction. mRNA expression was normalized to beta-actin mRNA and expressed as a relative value to the control group.

### Statistics

Normality of data was determined using the Shapiro–Wilk test and equality of variance verified using Brown-Forsythe test, and all data presented normal distribution. Statistical analyzes were performed using GraphPad Prism® software (GraphPad Software Inc, La Jolla, USA). Results were considered significant when *p* < 0.05 and data are shown as means ± standard error of the mean (SEM). Differences between two groups were evaluated by using unpaired Student's test, and differences between more than two groups were evaluated by using ANOVA.

## Results

### Basic characterization of kapBk rats

The DNA construct coding for an engineered fusion protein controlled by the kidney androgen-regulated protein (Kap) promoter was inserted into the genome of Sprague-Dawley rats (overview see in Figure 1A) to elicit BK overproduction specifically in the proximal convoluted tubule. This promoter is androgen dependent and was shown to be specific for proximal convoluted tubules (20). Adult male rats expressed the recombinant protein in the kidney (Figure 1B). Transgenic female rats cannot activate the Kap promoter and can be used as control. Low expression in the lung was also detected. Since BK in lung is very quickly inactivated as described before (30), the effect in lung is probably minimal. Figure 1C presents an image of the immunohistochemical detection of the fusion protein in kidney sections using an antibody directed against mouse immunoglobulin G2b, which is part of the engineered protein (Figure 1A), confirming its specific expression in proximal tubules of KapBK rats.

### KapBk rats exhibit polyuria, diluted urine, and excrete more Na^+^, but are able to control urine volume under water deprivation

**Figure 2:**
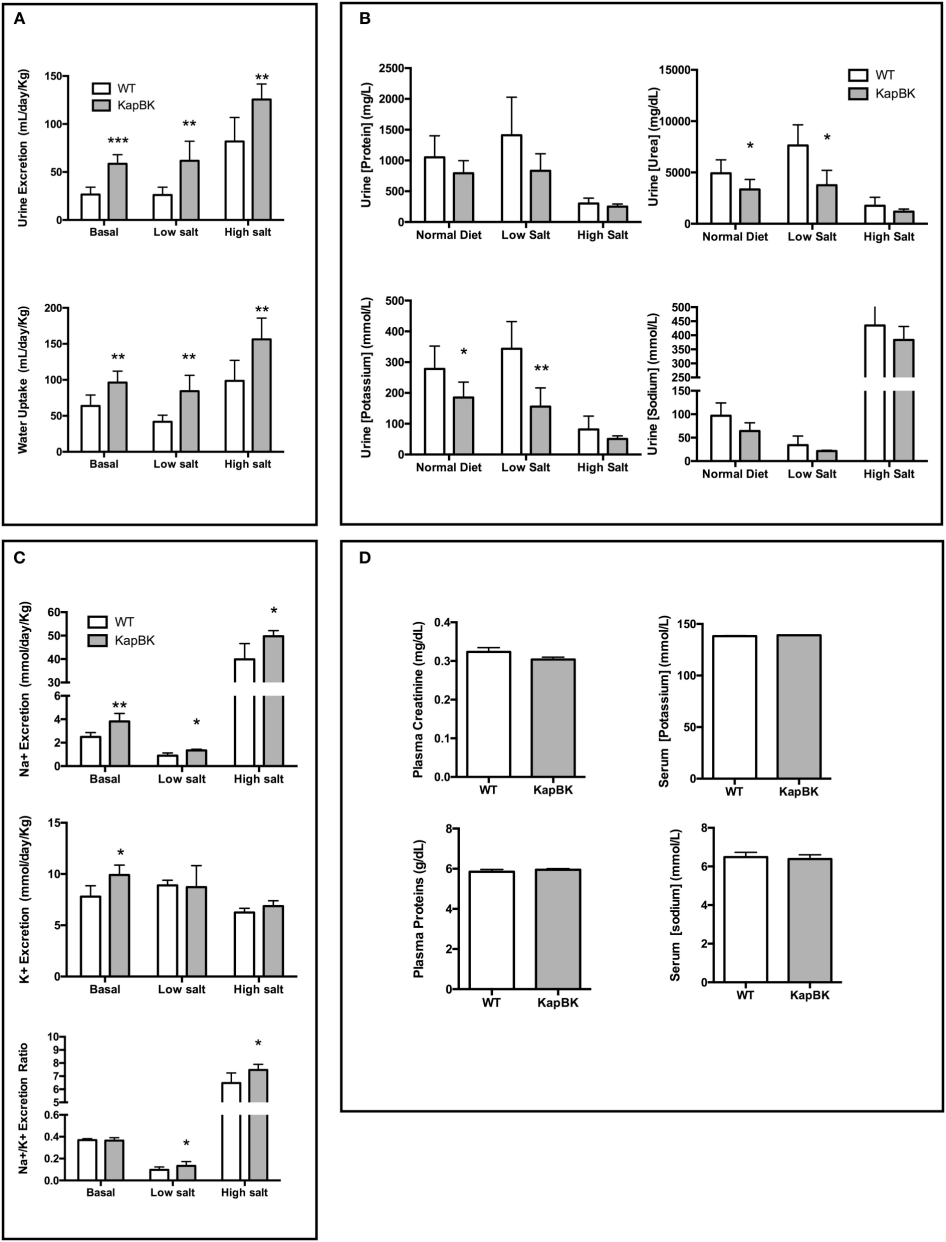
Urine and blood parameters. The animals were submitted to diets containing different concentrations of sodium. After 10 days with each diet urine was collected in metabolic cages. KapBK rats secrete more urine and drink more water **(A)** and the urine has lower concentrations of total proteins, urea, potassium and sodium concentrations. **(B)** Total sodium and sodium/potassium ratios are increased. **(C)** Plasma concentrations of total proteins, creatinine, sodium, and potassium were unchanged in rats fed with standard diet. **(D)** Data presented as mean ± SEM. *p < 0.05, **p < 0.01, ***p < 0.001 vs. WT, *n* = 6.

The overexpression of BK in renal proximal tubules causes polyuria which is compatible with other experiments infusing BK in anesthetized animals (17). The increase in urine excretion was present when rats were feeding a standard chow as well as with diets containing low and high amounts of salt (Figure 2A). The increased diuresis is compensated by reflex polydipsia (Figure 2A). In the case of high salt diet, KapBK rats need a smaller adaptive increase in urinary volume in order to excrete the excess of ingested salt. Furthermore, the urine of KapBK rats is diluted with regards to several other components like protein content, sodium, potassium, and urea suggesting reduction of water reabsorption (Figure 2B). Analyzing daily sodium and potassium excretion, we noted an increased sodium excretion and an increased Na^+^/K^+^ excretion ratio (Figure 2C) in agreement with previous studies (7, 15, 31, 32). No changes were observed regarding plasma levels of proteins, creatinine, sodium or potassium (Figure 2D), and blood cell count (Table S1) and only body composition analysis (Figure S1) showed a trend to increased water content.

**Figure 3:**
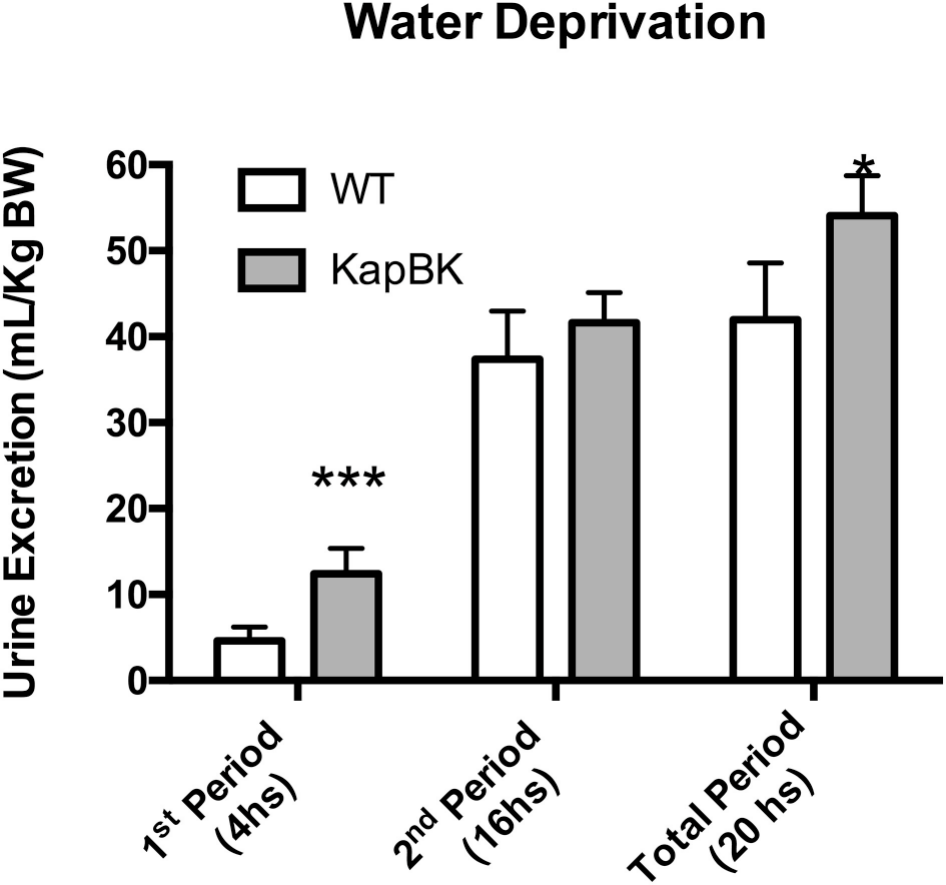
Urine excretion under water deprivation. Under normal diet, the animals were submitted to water deprivation and showed adaptation in urine volume production over time. Data presented as mean ± SEM. *p < 0.05, ***p < 0.001 vs. WT, *n* = 6.

Under 20-h water deprivation, KapBK rats present diuresis only in the first 4 h of the experiment. Then they start to produce the same amount of urine as the controls, showing that these rats are able to adapt to water deprivation avoiding severe dehydration (Figure 3). These results demonstrate that KapBK rats are a useful model for analyses of the chronic effect of increased renal BK on water/electrolyte homeostasis.

### KapBk rats present a small increase in systolic blood pressure, increased heart rate, and decreased heart rate variability

**Figure 4:**
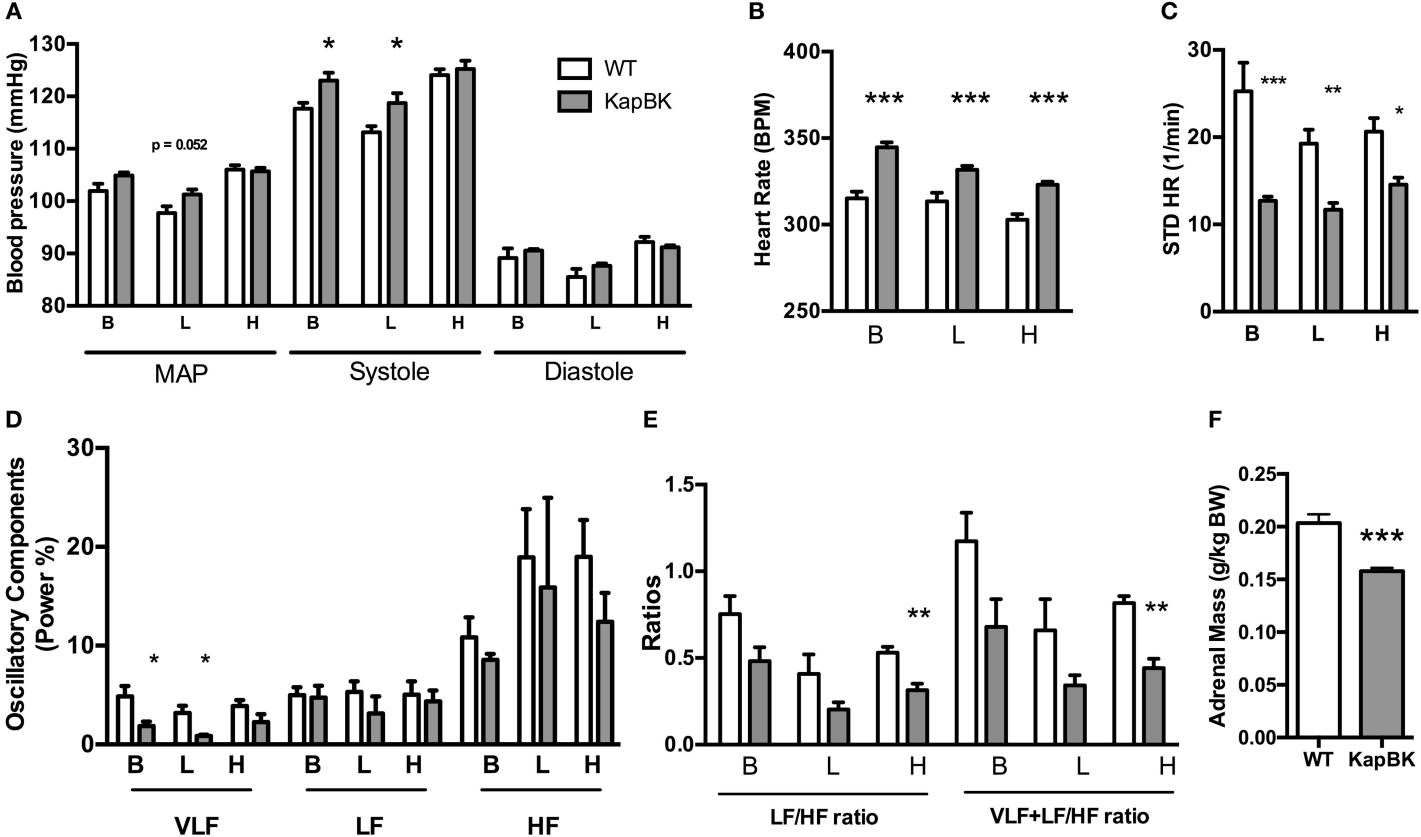
Cardiovascular and autonomic system. The blood pressure parameters were measured by telemetry after 10 days of normal (B), low salt (L), or high salt (H) containing diet. **(A)** Mean arterial pressure (MAP), systolic blood pressure (systole) and diastolic blood pressure (diastole). **(B)** Heart rate under normal diet (basal, B) and low (L) and high (H) salt diets. **(C)** Heart rate variability (HRV; STD HR = standard deviation of Heart Rate). **(D)** Power (%) of oscillatory components (VLF, very low frequency; LF, low frequency and HF, high frequency). **(E)** Ratios of oscillatory components. **(F)** Adrenal gland mass normalized to body weight. Data presented as mean ± SEM. *p < 0.05, **p < 0.01, ***p < 0.001 vs. WT, *n* = 6.

Chronically increased BK in renal proximal tubules of rats did not change MAP but increased SBP (Figure 4A) as detected by telemetric measurement (Figure S2). However, the cardiovascular system underwent several alterations in the adaptive response to the polyuria. Increased heart rate (Figure 4B) was observed, accompanied by decreased HRV (Figure 4C) and a decreased low-frequency oscillatory component (that can be a signal of changes in RAS) (Figure 4D). The LF/HF ratio (Figure 4E) was decreased in KapBK rats indicating decreased sympathovagal balance on the heart contrasting the increased heart rate. The relative and absolute masses of the adrenal glands were reduced in KapBK rats suggesting reduction of adrenergic activation (Figure 4F).

### Chronic BK excess in kidney causes cardiac hypertrophy and increased cardiac output, contrasting with smaller renal artery blood flow, and normal glomerular filtration rate

**Figure 5:**
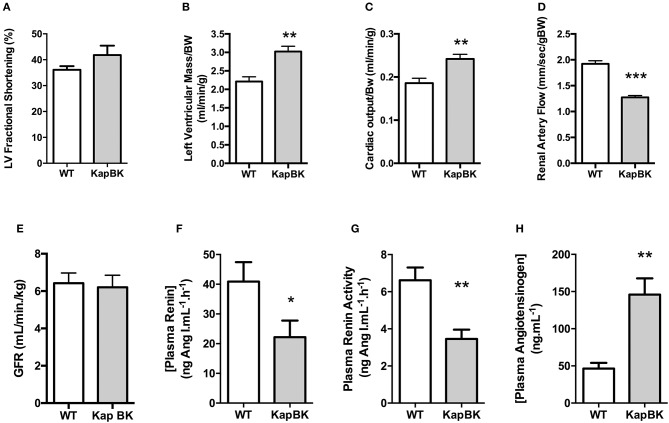
Cardiac and renal function, renin-angiotensin system. **(A)** left ventricular fractional shortening; **(B)** left ventricular mass; **(C)** normalized cardiac output; **(D)** renal artery flow; **(E)** glomerular filtration rate (GFR) as estimated by creatinine clearance **(F)** plasma renin concentration; **(G)** plasma renin activity; **(H)** plasma angiotensinogen concentration. Data presented as mean ± SEM. *p < 0.05, **p < 0.01, ***p < 0.001 vs. WT, *n* = 6.

Probably as a consequence of the increased heart rate, cardiac hypertrophy develops in older KapBK rats (6 months old) as evidenced by increased left ventricular fractional shortening, left ventricular mass, and cardiac output (Figures 5A–C). Despite the increased cardiac output, KapBK rats have reduced renal artery blood flow (Figure 5D) suggesting high pressure in the glomerular capillary system. The activity of the systemic RAS is changed (Figures 5F–H) with decreased plasma renin activity and concentration (2-fold) and increased concentration of plasma angiotensinogen (3-fold). Connecting the low blood flow in the renal artery with the polyuria, we found normal GFR as estimated by creatinine clearance (Figure 5E).

### Chronic overexpression of BK in kidney increases local angiotensinogen and decreased epithelial sodium channel (SCNN1, alpha, and gamma subunits) and AVPR1A mRNA expression

**Figure 6:**
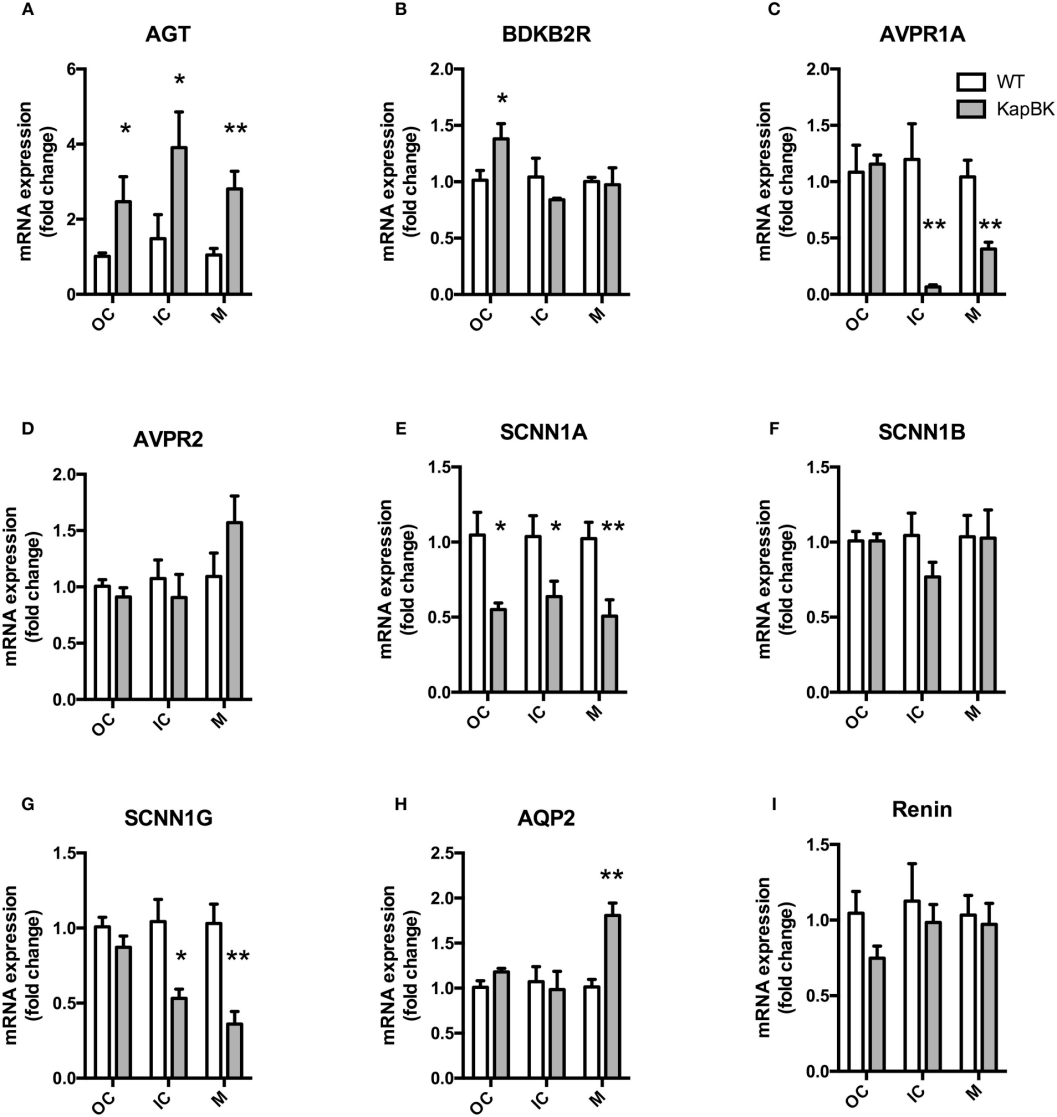
Renal gene expression: Total RNA was extracted from outer (OC) and inner (IC) cortex, and medulla (M) of kidney. **(A)** AGT, angiotensinogen; **(B)** BDKB2R, bradykinin B2 receptor; **(C)** AVPR1A, vasopressin V1A receptor; **(D)** AVPR2, vasopressin V2 receptor; **(E–G)** SCNN1-A, B, and G, subunits alpha, beta, and gamma of the Epithelial Sodium Channel (ENaC); **(H)** AQP2, aquaporin 2; **(I)** renin. All data were normalized for WT OC group and are shown as relative fold change. Data presented as mean ± SEM. *p < 0.05, **p < 0.01 vs. WT, *n* = 5.

We analyzed the mRNA expression of selected genes by qPCR in the following regions of the kidney: Fragments of outer cortex (OC), inner cortex (IC), and medulla (M) (Figure 6). Increased angiotensinogen expression was observed in all parts of kidney (Figure 6A), showing that the local RAS is activated in KapBK rats. We also found increased expression of B2R in OC (Figure 6B), probably due to the increased BK levels that were shown to stimulate the expression of B2R by Ricciardolo et al. in other tissues (33). The reduced expression of vasopressin V1A receptor (AVPR1A, Figure 6C) can explain the diluted urine although vasopressin V2 receptor (AVPR2) expression remained unchanged (Figure 6D). Increased sodium excretion can be related to reduced expression of alpha and gamma subunits of the epithelial sodium channel (ENaC, SCNN1, Figures 6E–G). The increased aquaporin 2 (AQP2) expression in medulla after water deprivation is part of the mechanism involved in the adaptation against severe dehydration (Figure 6H). No changes were observed in renal mRNA expression of renin (Figure 6I).

## Discussion

Acute infusion of bradykinin in the kidneys causes polyuria inhibiting vasopressin activity in collecting tubules (17, 18, 34) and increasing sodium excretion (1, 7, 9, 10, 15, 32, 35). However, the consequences of chronic BK excess in the kidneys are still incompletely studied (36, 37). In the present study a new animal model producing an excess of BK in the proximal tubules was generated. The overproduction of BK in these rats starts from puberty and is maintained during the remainder of life, allowing studies about long-term effects of BK in kidney. For better understanding of these chronic effects, the animals were submitted to three diets with different salt concentrations. Changes in renal and cardiovascular functions as well as in the renin-angiotensin and KKSs were assessed. Additionally, the expression of several genes was evaluated in different parts of the kidney. The main results showed that the chronic BK excess in the kidneys causes cardiac hypertrophy, changes in the regulation of the local, and systemic renin angiotensin systems, increased heart rate and cardiac output, decreased heart rate variability, maintenance of GFR despite of reduced blood flow in the renal artery, and changes in gene expression in kidney, mainly increasing angiotensinogen and decreasing vasopressin V1A receptor mRNA levels. Moreover, there was no change in MAP, in agreement with the described acute models of BK excess in the kidney (17, 36).

The results confirmed that the overexpression of BK in the kidney of the animals was successfully achieved. Although we could not measure BK levels directly, this conclusion is supported by genotyping, RT-PCR, immunohistochemistry and also by the concordance of physiological changes observed in the present study with previous literature, mainly polyuria, increased sodium excretion and the diluted urine of the transgenic animals. These effects were observed after acute BK treatment in kidney (17) but also in studies searching for chronic effects (36). Furthermore, the DNA construct was designed to be expressed in proximal tubules and to secrete BK into the extracellular space, based on the signal peptide present in the expressed protein and the ubiquitous expression of furin, and it is well-established that intraluminally generated kinins in kidney exert autocrine and paracrine actions on distal tubular cells, favoring natriuresis and diuresis (32) as observed in the present study. In addition, interstitially released BK contributes to the regulation of cortical and medullary blood flow (38), as was also observed in KapBK rats and will be discussed below. These results confirm that the novel rat model is appropriate to study chronic excess of BK in kidney.

Although BK is known to dilate vessels and promote sodium excretion and polyuria, very few experiments show a sustained alteration in MAP by BK. Different methods were used to test the potential hypotensive effect by infusing BK in kidney, but BP normally returned to normal values after an acute decline (37, 39, 40). Moreover, it was also observed that knockout mice for either kinin receptor, B1 and B2, as well as double knockout mice are normotensive (41–45), although they can react differently than controls in relation to BP control when submitted to challenges such as high-salt diet. Only general overexpression of tissue-kallikrein in transgenic rats leads to a small but significant reduction in MAP probably by the permanent rise of BK in several tissues (42). Although KapBK rats presented similar MAP as the controls, we observed several changes in the cardiovascular system concerning BP regulation. The local renal RAS seems to be activated with increased expression of angiotensinogen. This increased intrarenal RAS activity is also evidenced by the diminished renal artery blood flow since AngII is known to cause vasoconstriction of both afferent and efferent glomerular arterioles increasing intraglomerular pressure (46). On the other hand, the observed reduction of plasma renin concentration and activity suggests a reduction in systemic RAS activity. Despite the severe reduction in renal artery blood flow in KapBK rats, GFR does not change. The reduction in glomerular blood flow seems to be a mechanism to maintain normal GFR which is also observed in AVPR1A knockout mice (47).

It is well established that BK inhibits vasopressin-induced water flow in the collecting duct *in vitro* (18, 34) and *in vivo* via B2R (17). The present study shows that after several months of chronic overexpression of BK in kidney the rats still present polyuria. The dilution of all urinary components indicates increased water content suggesting decreased water reabsorption. This is compatible with the inhibition of vasopressin activity as previously described both *in vitro* and *in vivo* in acute models of BK infusion in the kidney (17, 18, 34). However, the present study shows a marked decrease in the expression of vasopressin V1A receptors in the parts of the kidney that are rich in distal nephron structures, but no change in V2 receptor expression, showing that vasopressin resistance can be regulated directly via gene expression of V1A receptors. Previous studies showed that the effect of BK on inhibition of vasopressin activity is dependent on prostaglandin formation (and can be blocked with indomethacin) and independent of increased calcium concentration (17). This suggests that the increase in endogenous prostaglandin synthesis by B2R activation antagonizes vasopressin-stimulated cAMP generation and reduces vasopressin-induced water flow in the collecting duct (18, 34). The new observation that decreased expression of vasopressin V1A receptors is related to these effects opens new doors to understand the underlying mechanisms. Further gene expression studies are warranted using acute models of excessive BK supply to elucidate whether the changed AVPR1A expression found here is indeed a chronic effect, or is also part of acute BK effects on vasopressin-induced water flow in the kidney. Favoring that vasopressin receptors mediate the effects of chronic BK excess, AVPR1A knockout mice exhibited a similar phenotype as KapBK rats concerning urine volume and composition (48). Accordingly, polyuria in KapBK rats seems to be an effect caused in the distal nephron by reduction of AVPR1A receptors as evidenced in the mRNA expression analysis. Aoyagi et al. showed that polyuria observed in AVPR1A knockout mice was associated with decreased water reabsorption and not with increased GFR, as observed in KapBK rats. They also described a lower renin concentration in plasma of AVPR1A knockout mice again agreeing with KapBK rats (14). Furthermore, Yasuoka et al. noted that in water restriction conditions AVPR1A knockout mice also increase renal AQP2 expression as observed in KapBK rats (47). All these similarities suggest that the consequences of increased BK in kidney are due to the altered regulation of AVPR1A expression in KapBK rats.

To localize gene expression to certain structures of the kidney, we used RNA originating from three different regions of the kidney, called here: the “outer cortex,” the more external part of the kidney cortex containing mainly the proximal structures of the nephrons; the “inner cortex,” containing all parts of the nephrons including small fractions of the distal nephrons; and the “medulla,” containing mainly collecting ducts and loops of Henle. One main result of this expression study was the reduction of alpha and gamma subunits expression of ENaC mainly in parts of the kidneys containing distal nephron structures. Again, these new data suggest that the inhibition of ENaC activity by BK infusion can be induced by gene expression regulation. It is well-established that the increase in UNaV by BK is due to the inhibition of ENaC (7, 15, 32). Other authors have shown inhibition of sodium reabsorption by BK (7, 31, 49). Zaika et al. presented a related effect on SCNN1 in an experiment *in vitro* (15), showing that BK decreases the probability of SCNN1 channel opening. In the present study, the ENaC activity was not measured since it was well-demonstrated previously (7, 17). However, the measurements of Na^+^, Cl^−^, and K^+^ in urine indicated that only Na^+^ showed increased excretion in KapBK rats, suggesting that the inhibition of Na^+^ reabsorption is not due to cotransporters such as Na/K-ATPase, thiazide-sensitive sodium chloride co-transporter (NCCT) or sodium-potassium-chloride co-transporter (NKCC2), all of them important sodium transporters in the apical membrane of different regions of the nephrons (50). Together with previous studies demonstrating that BK inhibits only ENaC (7, 15, 17, 32), the here presented reduction of the gene expression of ENaC subunits, and the urine concentration of the excreted ions together indicate that the observed natriuresis in KapBK rats is caused by reduction of ENaC, at least partially on the level of gene expression.

Although vasopressin sensitivity is reduced in KapBK rats by decreased AVPR1A expression, expression of AVPR2 is similar to that of control animals. Accordingly, AQP2 mRNA expression is increased in renal medulla of KapBK rats after water deprivation. AVPR2 is the main regulator of AQP2 translocation to the apical membrane providing a strong mechanism of water reabsorption in the presence of increased vasopressin levels. Both, the induction of AQP2 expression and normal expression of AVPR2 explain why KapBK rats can concentrate urine under water deprivation, and fit with observations in AVPR1A knockout mice, which also can concentrate urine under this condition (14). Therefore, we speculate that KapBK rats are equipped with a reduced sensitivity to basal vasopressin levels, due to low AVPR1A expression, but they respond adequately to increased level of vasopressin, since there is a normal AVPR2 expression and an inducible AQP2 expression in kidney.

The increased renal BK expression affects the heart as evidenced by increased heart rate and contractility in KapBK rats, leading to an augmented cardiac output and even cardiac hypertrophy when KapBK rats get older. These are typical phenomena of cardiac sympathetic stimulation, but we found decreased LF/HF ratio and a smaller mass of the adrenal gland suggesting a reduction in the sympathovagal balance. This discrepancy may at least partially be related to the differential regulation of the sympathetic innervation of different organs.

Taken together, our results show that chronic intrarenal overexpression of BK exerts similar alterations in renal physiology as known from acute models of intrarenal BK infusion. However, also unexpected effects appeared in the chronic model showing that the new transgenic KapBK rats are suitable for studying chronic actions of BK in the kidney. These new effects of intrarenal BK include changes in RAS regulation, decreased renal vasopressin sensitivity based on decreased V1A receptor expression and reductions in cardiac rhythm variability, augmentation in heart rate and cardiac output and consequently cardiac hypertrophy without changes in MAP. These data have implications for the concept that B2 receptor agonists may have a beneficial effect in controlling hypertension. It is already well-established that ACE inhibitors exert their main effects by reducing angiotensin II, but in addition, much of the protective effects on the kidneys are associated with the increase of the BK concentrations in this organ (51–54). In the present study, the chronic increase of renal BK happened in the presence of an active RAS, since there is no ACE inhibition. The present data suggest even an increased local RAS activity, based on increased angiotensinogen expression and decreased renal artery blood flow. The systemic RAS activity, on the other hand, seems to be decreased based on decreased plasma renin concentration and activity. Under these conditions, novel adaptations and physiological effects of BK have been reported here, which should be followed up in the case of administration of B2 receptor agonists as experimental treatment in patients without ACE inhibitor treatment.

## Author contributions

CB conducted the physiological analyses, collecting, organizing and analyzing all data, and writing of manuscript. IS performed the molecular analyses. GS conducted urine analyses. FR genotyped the rats. PX cloned the DNA vector to generate the animals. EP made the microinjections to generate the animals. IL performed the radioimmunoassay for RAS measurements. RP analyzed telemetric data. AH conducted echocardiography and its analysis. MT conducted surgeries and telemetric data analysis. SB performed immunohistochemistry of the kidneys. NA helped to design the experiments, and with the drafting and revision of the manuscript. RA helped with data interpretation, drafting, and revision of the manuscript. JP helped with data interpretation, drafting, and revision of the manuscript. MB conceived and designed the work, drafted, and revised the manuscript.

### Conflict of interest statement

The authors declare that the research was conducted in the absence of any commercial or financial relationships that could be construed as a potential conflict of interest.
