# Supplementary material for: Chronic Overexpression of Bradykinin in Kidney Causes Polyuria and Cardiac Hypertrophy
Source: Front Med (Lausanne). 2018 Dec 3;5:338. doi: 10.3389/fmed.2018.00338 (PMC6287039; doi:10.3389/fmed.2018.00338)
Supplement: Supplementary file 1 [file Data_Sheet_1.PDF]

frontiers

Supplementary Material

Chronic Overexpression of Bradykinin in Kidney Causes Polyuria and Cardiac Hypertrophy

***Carlos C Barros[1]; Ines Schadock[2,3]; Gabin Sihn[3]; Franziska Rother[3]; Ping Xu[3]; Elena Popova[3]; Irina Lapidus[3]; Ralph Plehm[3]; Arnd Heuser[3]; Mihail Todiras[3]; Sebastian Bachmann[5]; Natalia Alenina[3,6]; Ronaldo C Araujo[2], Joao B Pesquero[2]; Michael Bader[3,5-9].**

*** Correspondence:** Carlos C Barros: barrossccpel@gmail.com

1 Supplementary Figures and Tables

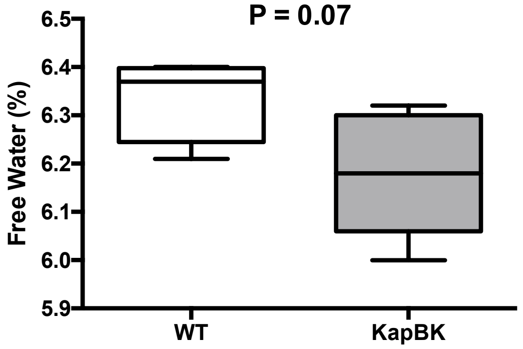

Figure S1: Free water analysis. Data obtained by body composition analysis and presented as mean ± SEM, n = 6.

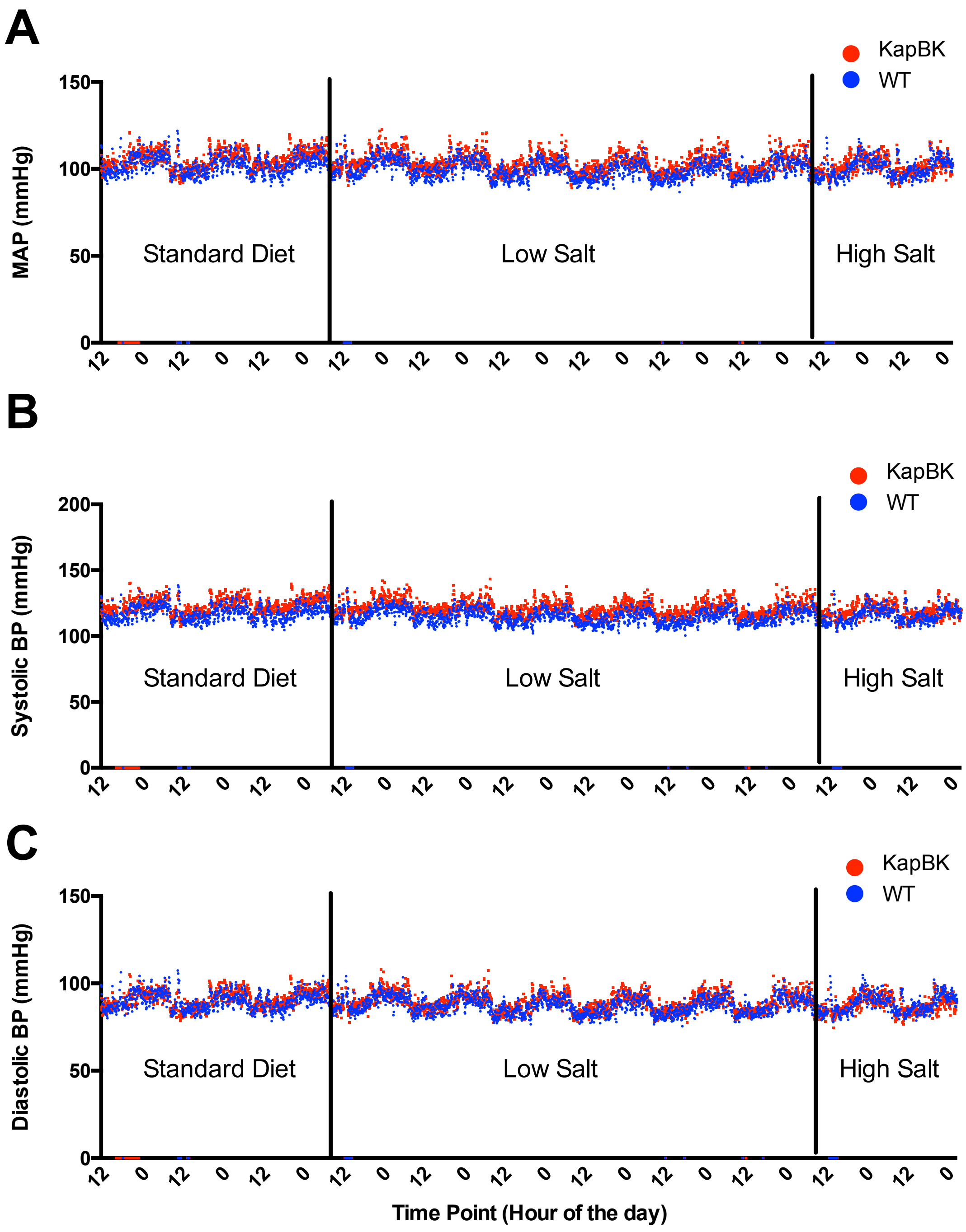

Figure S2: Circadian blood pressure rhythm recorded by telemetry. A) Mean Arterial Pressure (MAP); B) Systolic Blood Pressure; C) Diastolic Blood Pressure. The graphics show the last days of each 10 days periods after surgery or introduction of new diet. The vertical bars separate data from rats fed with different diets and represent time break points. Each dot represent the mean of the group recorded at each 5 minutes. n=6.

Table S1: Blood cell counts of KapBK rats and controls (n=2)

Blood cell counts	WT	BK	t-test
WBC - white blood cell count (K/µL)	16.37 ± 1.124	15.35 ± 0.087	0.312
RBC - red blood cell count (M/µL)	7.11 ± 0.221	6.98 ± 0.026	0.463
HGB - hemoglobin (g/dL)	17.97 ± 0.551	16.35 ± 0.087	0.079
HcT - hematocrit (%)	39.03 ± 1.589	36.85 ± 0.087	0.163
MCV - mean corpuscular volume (fL)	55.00 ± 1.000	53.00 ± 2.870	0.075
MCH - mean corpuscular hemoglobin (pg)	25.23 ± 0.115	23.45 ± 0.260	0.107
MCHC - mean corpuscular hemoglobin concentration (g/dL)	46.07 ± 0.808	44.40 ± 0.520	0.081
RDW - red cell distribution width (%)	11.47 ± 0.058	11.05 ± 0.087	0.053
Plt - platelet count (K/µL)	598.00 ± 190.139	626.50 ± 99.593	0.860
MPV - mean platelet volume (fL)	5.20 ± 0.173	5.25 ± 0.087	0.735
%Lym - lymphocytes (%)	72.03 ± 1.429	66.90 ± 2.078	0.065
%Mo - monocytes (%)	4.20 ± 0.173	5.90 ± 0.866	0.070
%GRA - granulocytes (%)	23.77 ± 1.429	27.20 ± 1.212	0.063
LYM - lymphocytes (K/µL)	11.73 ± 0.702	10.15 ± 0.260	0.060
MON - monocytes (K/µL)	0.63 ± 0.058	0.85 ± 0.087	0.072
GRA - granulocytes (K/µL)	4.00 ± 0.436	4.35 ± 0.260	0.383
